# Similar early characteristics but variable neurological outcome of patients with a de novo mutation of *KCNQ2*

**DOI:** 10.1186/1750-1172-8-80

**Published:** 2013-05-22

**Authors:** Mathieu Milh, Nadia Boutry-Kryza, Julie Sutera-Sardo, Cyril Mignot, Stéphane Auvin, Caroline Lacoste, Nathalie Villeneuve, Agathe Roubertie, Bénédicte Heron, Maryline Carneiro, Anna Kaminska, Cécilia Altuzarra, Gaëlle Blanchard, Dorothée Ville, Marie Anne Barthez, Delphine Heron, Domitille Gras, Alexandra Afenjar, Nathalie Dorison, Dianne Doummar, Thierry Billette de Villemeur, Isabelle An, Aurélia Jacquette, Perrine Charles, Julie Perrier, Bertrand Isidor, Laurent Vercueil, Brigitte Chabrol, Catherine Badens, Gaétan Lesca, Laurent Villard

**Affiliations:** 1 INSERM, UMR_S 910 Faculté de médecine, Boulevard jean MOULIN F13005, Marseille, France; 2 APHM, Service de neurologie pédiatrique, CHU Timone, Marseille, France; 3 Hospices civils de Lyon. Laboratoire de génétique, Hôpital Edouard Herriot. Bron, Lyon, France; 4 APHP, Unité Fonctionnelle de Génétique Médicale, Département de Génétique, Groupe Hospitalier Pitié-Salpêtrière, Paris, France; 5 Centre de Référence des Déficiences Intellectuelles de Causes Rares, Paris, France; 6 APHP. Service de Neuropédiatrie, Hôpital Armand Trousseau, Paris, France; 7 APHP. Service de neuropédiatrie, Hopital Robert Debré, Paris, France; 8 APHM. Département de Génétique Médicale et Biologie Cellulaire CHU Timone, Marseille, France; 9 CHU Montpellier. Service de neuropédiatrie, Montpellier, France; 10 INSERM U1051, INM Montpellier, Montpellier, France; 11 APHP. Service de neurophysiologie clinique Hôpital Necker, Paris, France; 12 CHU Besancon. Service de neuropédiatrie, Besancon, France; 13 Hospices civils de Lyon, Service de neuropédiatrie. HFME. Bron, Lyon, France; 14 CHU de Tours. Service de neuropédiatrie, Beranger, France; 15 APHP. Groupe hospitalier Pitié Salpétrière. Service de neurologie, Paris, France; 16 CHU de Nantes. Service de pédiatrie, Nantes, France; 17 CHU de Nantes. Service de génétique médicale, Nantes, France; 18 CHU de Grenoble. Service d’électrophysiologie clinique, Grenoble, France; 19 Aix Marseille Université, Faculté de Médecine, Marseille, France

**Keywords:** Epilepsy, Genetics, KCNQ2, Encephalopathy

## Abstract

**Background:**

Early onset epileptic encephalopathies (EOEEs) are dramatic heterogeneous conditions in which aetiology, seizures and/or interictal EEG have a negative impact on neurological development. Several genes have been associated with EOEE and a molecular diagnosis workup is challenging since similar phenotypes are associated with mutations in different genes and since mutations in one given gene can be associated with very different phenotypes. Recently, de novo mutations in *KCNQ2*, have been found in about 10% of EOEE patients. Our objective was to confirm that *KCNQ2* was an important gene to include in the diagnosis workup of EOEEs and to fully describe the clinical and EEG features of mutated patients.

**Methods:**

We have screened *KCNQ2* in a cohort of 71 patients with an EOEE, without any brain structural abnormality. To be included in the cohort, patient’s epilepsy should begin before three months of age and be associated with abnormal interictal EEG and neurological impairment. Brain MRI should not show any structural abnormality that could account for the epilepsy.

**Results:**

Out of those 71 patients, 16 had a de novo mutation in *KCNQ2 (23%)*. Interestingly, in the majority of the cases, the initial epileptic features of these patients were comparable to those previously described in the case of benign familial neonatal epilepsy (BFNE) also caused by *KCNQ2* mutations. However, in contrast to BFNE, the interictal background EEG was altered and displayed multifocal spikes or a suppression-burst pattern. The ongoing epilepsy and development were highly variable but overall severe: 15/16 had obvious cognitive impairment, half of the patients became seizure-free, 5/16 could walk before the age of 3 and only 2/16 patient acquired the ability to speak.

**Conclusion:**

This study confirms that *KCNQ2* is frequently mutated de novo in neonatal onset epileptic encephalopathy. We show here that despite a relatively stereotyped beginning of the condition, the neurological and epileptic evolution is variable.

## Background

*KCNQ2* encodes a channel subunit carrying the neuronal Im current whose inherited mutations were first described in autosomal dominant benign familial neonatal epilepsy (BFNE, OMIM#121200) [1-3]. Patients affected by a BFNE displayed stormy phase of motor seizures during the neonatal period, lasting 2 to 6 weeks in average. Interictal EEG was normal or slightly modified [4]. Subsequently, seizure frequency quickly decreased and the vast majority of patients became seizure free before the age of three months [5]. Motor and cognitive outcome were usually normal. Recently, de novo mutations of *KCNQ2* have been described in early onset epileptic encephalopathies (EOEEs; OMIM#613720) [6-8]. EOEEs are a group of devastating epilepsies beginning before three months of age, with frequent seizures and abnormal interictal EEG leading to a rapid deterioration of motor, cognitive and sensori-neuronal functions. Patients carrying de novo *KCNQ2* mutations displayed abnormal interictal EEG that could reveal multifocal spikes or a suppression-burst pattern, and all had poor neurological outcome [7,8]. This dramatic form of *KCNQ2*-related epilepsy, with very poor neurological outcome, was unexpected. In order to assess the importance of *KCNQ2* screening for the molecular diagnosis of early onset epilepsies, and mostly to describe the outcome of the sporadically mutated patients, we have analyzed a cohort of 71 patients with an early onset, severe epilepsy, without any familial history of epilepsy.

## Methods

This study was approved by CPP Sud Méditerannée (Comité de protection des personnes). Seventy one patients were included in a cohort of subjects who displayed an early onset epileptic encephalopathy. All the patients or their parents gave their informed consent to join the cohort. Inclusion in the cohort was decided according to the following criteria; (1) epilepsy onset within the first 3 months of age; (2) abnormal interictal EEG (3) brain MRI without obvious cortical malformation or hypoxic lesion; (4) normal metabolic screening (exclusion of nonketotic hyperglycinemia, hyperammonemia, urea cycle defect, organic aciduria, hyperlactacidemia, pyridoxine-dependent and pyridoxal-dependent seizures); (5) No mutation of *STXBP1*, a major gene involved in early onset epileptic encephalopathy with or without suppression-burst [9]; (6) No mutation of ARX [10] in male patients (n=35); (7) patients must be regularly followed till now. All the girls that displayed early onset epileptic spasms and/or tonic seizures without any suppression-bursts were tested for *CDKL5* (n=36). The epilepsy began during the neonatal period for 47/71 patients, the EEG showed a suppression-burst or discontinuous traces in 33 of them (Groupe A), and multifocal spikes in the remaining 14 (Groupe B). Epilepsy began between 1 and 3 months for the 24 patients of groupe C. The 18 coding exons (including alternative exons) of *KCNQ2* were sequenced. Primer sequences are available upon request. The identified mutations were numbered according to the *KCNQ2* reference sequence NM_172107.2.

## Results and discussion

**Figure 1:**
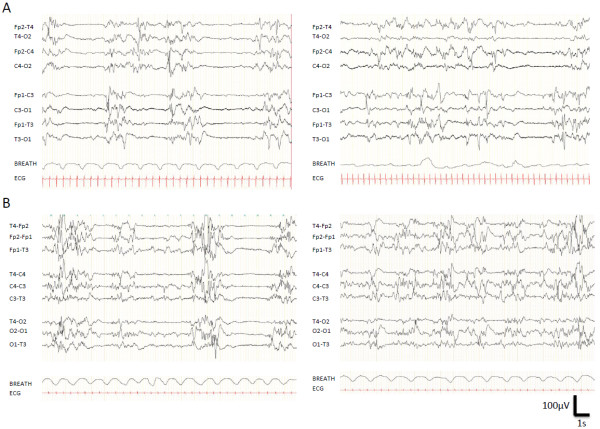
**Representative early EEGs of patients carrying a de novo KCNQ2 mutation. A**. Interictal EEG (Day 3, patient 1), showing a typical suppression-burst pattern, with burst of spikes and slow waves alternating with periods of electric silence (left panel). Sometimes, the burst should be much longer than the periods of suppression, leading to a discontinuous pattern (Right panel). **B**. EEG displaying the same features (Patient 12, suppression-burst in left panel, discontinuous pattern in right one), with a very different outcome (normal development at 5 years old, see Table 1).

We found heterozygous mutations in *KCNQ2* in 16/71 patients (Table 1). All of them have occurred *de novo*. Typically, the first seizure was observed before the 5^th^ day of life (n=14/16), taking the form of clonic and/or tonic seizures resembling those observed in BFNE (12/16). These seizures were very frequent, rapidly leading to obvious neurological impairment before the end of the first week (10/16). Eight patients carrying a *KCNQ2* mutation were initially diagnosed with an Ohtahara syndrome, with a typical suppression-burst pattern on the EEG (Table 1, Figure 1). The first EEG did not show any suppression-burst pattern, but discontinuous traces in the remaining patients (Table 1, Figure 1). In three cases, EEGs evolved into a hypsarythmic pattern, but the majority quickly developed into a continuous pattern with multifocal asynchronous spikes and/or slowing of the traces (13/16). The outcome of epilepsy was highly variable: 9/16 patients became seizure free during the follow-up, 6 of them before the end of the first year of life, while 7/16 patients were still epileptic, three of them had only myoclonic jerks, two of them had recurrent generalized tonic clonic seizures and two had focal seizures (Table 1). Fifteen patients had obvious developmental delay: 4/15 could walk but 3/4 had no language and 1/3 had autistic features; 11/15 were profoundly impaired with poor or absent head control and eye contact (8/15) or global/axial hypotonia with poor or absent hand use (3/15). One patient had a good evolution with normal neurological evaluation at age 6. The initial brain MRI was normal or showed very slight and transitory brain signal abnormalities in 12/16 patients, while in 3 patients, abnormal signal intensity were found, as previously described [7] (Table 2). Only one patient had extra-neurological features: congenital left hip luxation and cleft palate (patient 2). Fifteen patients had a mutated *KCNQ2* in the group A (45%, n=33), one patient had a mutation of *KCNQ2* in the group B (7%, n=14) and none of the patient had a KCNQ2 mutation in the group C. Thus, KCNQ2 was mutated in half of patients with a neonatal onset epileptic encephalopathy and an EEG showing either discontinuous or suppression-burst pattern. Here, we confirm that, besides well described entity BFNE, *KCNQ2* mutations can also be associated with severe epileptic and cognitive phenotypes defining early onset epileptic encephalopathies [7]. Hence, it should be considered in the diagnosis workup of neonatal onset epilepsies especially those beginning during the first week of life with stormy clonic and/or tonic seizures, whatever the presence or absence of a familial history. If cognitive outcome is relatively reliable and good in familial cases of BFNE [5], it is not the case in sporadic ones, where neurological outcomes range from dramatic to normal. We did not find any relationship between the initial history of the epilepsy and the severity of outcome. For example, patients 1 and 12 had relatively similar features at the beginning and displayed very different outcomes (Tables 1, 2 and Figure 1). Since *KCNQ2* is now implicated in various forms of epilepsies, from the most benign to the most dramatic, additionnal data on phenotype/genotype correlations would be particularly relevant. Interestingly, none of the mutation reported in neonatal epileptic encephalopathies had previously been reported in BFNE, and the severe mutations that have been found in several patients (p.G290A [7], p.T274M and p.A294V(present study)) lead to relatively similar features in terms of initial EEG and development, however different in terms of evolution of the epilepsy. The different epileptic features in patients carrying the same mutation of KCNQ2 may be due to genetic modifiers or non genetic factors. Overall, this cohort of patients highlights the heterogeneous evolution of the neurological phenotypes associated with de novo heterozygous mutations in *KCNQ2*. This heterogeneity could be at least partially related to the impact of the mutations on the Im current. Analysis of the functional consequences of “benign” versus “severe” mutations in *KCNQ2* should be of paramount importance to better understand the molecular and cellular mechanisms involved in the emergence of an epileptic encephalopathy. This has recently been tested with two mutations of *KCNQ2* affecting the same residue in the S4 domain of the protein KV7-2 but associated with either a benign phenotype, or with a neonatal epileptic encephalopathy with severe drug-resistant seizures and neurocognitive delay, suppression-burst pattern at EEG, and distinct neuroradiological features [11]. The authors showed that, while both mutations destabilized the open state of the channel causing a reduction of the voltage sensitivity, the functional changes were more pronounced in the “severe” mutation than in the benign one. In both cases, the functional impairment could be fully restored by the neuronal Kv7 activator retigabine. This study suggested that the clinical disease severity may be related to the extent of the mutation-induced functional K+ channel impairment and set the preclinical basis for the potential use of Kv7 openers as a targeted anticonvulsant therapy to improve developmental outcome in neonates. However, since two patients carrying the same *KCNQ2* mutation do not have the same epileptic outcome, correlations between Im impairment and the severity of the encephalopathy should be made with caution. Other unknown factors may be involved in the occurrence of the epileptic encephalopathy. Moreover, the vast majority of the mutations we described here were localised on segment S6 and should have different consequences on Im current that those which have been previously described, affecting segment S4 [11]. These consequences still have to be studied. Overall, it is not known whether ongoing brain dysfunction that is observed in several patients is due to the Kv7-2 channelopathy or if it is a sequel of neonatal epilepsy. This question would be of paramount interest.

**Table 1:** KCNQ2 mutations and main features of the patients

	**Mutation**	**Seizure onset (days)**	**Initial seizure type**	**Seizure evolution**	**First EEG**	**Development (age at evaluation)**
**Patient 1**	c.C860A p.T287N	1	Clonic and tonic. Multiple seizures daily.	2 weeks: seizure offset.	Suppression-burst.	Poor eye contact, poor head control (9 months). Normal HC.
**Patient 2**	c.G523T p.V175L	15	Myoclonic jerks. No erratic myoclonus.	0-3 months: myoclonic jerks.	Discontinuous.	Deceased at 17 months.
					Bursts of polyspikes generalized or in the in central regions.	No eye contact, no head control. HC : 41cm.
				3–6 months: reflex audiogenic seizures.		
				6–12 months: epileptic spasms.		
				>12 months: myoclonic jerks.		
**Patient 3**	c.C926T p.A309V	3	Tonic, pallor, Multiple seizures daily.	0-24 months: multiple daily focal seizures. 2–5 years: 1 seizure/week. 7 years: epilepsy offset.	Suppression-burst.	Poor eye contact. Global hypotonia, unable to sit (2 years)
**Patient 4**	c.C821T p.T274M	2	Tonic and hypotonic. Epileptic spasms.	2 months: seizure free. Erratic intermittent myoclonus.	Suppression-burst.	Poor eye contact, no head control, global hypotonia (14 years). Normal HC.
					Right temporal, asymptomatic seizures.	
**Patient 5**	c.G715C p.G239R	2	Tonic and tonic-clonic, cyanosis.	2-6 weeks: Tonic and tonic-clonic seizures in clusters. 2 m: seizure stop	Poor activity. Prolonged periods of flatness of the traces. Generalized spikes predominating on the left hemisphere. Then suppression-burst.	Good eye contact. Sitting, hand use (10 months). Walking (22 months).
						No speech (4 y) Normal HC
**Patient 6**	c.C881T p.A294V	2	Left and right clonic jerks, facial cyanosis.	3 months: Seizure offset.	Suppression-burst.	Poor head control, unable to sit, no voluntary movement, no language (2 years).
**Patient 7**	c.C881T p.A294V	1	Isolated access of cyanosis. Then recurrent hypertonic posture.	7 months: epileptic spasms.	Suppression-burst.	Eye contact. Strabismus.
						No sit, no speech (11 y).
					Multiple focal seizures: tonic contractions of one or several limbs, cyanosis.	
				2–9 years: seizure-free.		
				> 9 years: monthly GTC seizures.		
**Patient 8**	c.T911C p.F304S	1	Tonic asymmetric.	2 months: multifocal seizures. 4 months: rhythmic jerks. 3 years: tonic seizures, cyanosis. 3-11y: Persistence of tonic seizures, cyanosis.	Bursts of multifocal spikes and periods of poorness of the activity.	Unable to sit, poor use of hands. No language. Feeding difficulties (gastrostomy, 11 years)
**Patient 9**	c.G566T p.G189V	3	Tonic.	0-6 months: multiple focal seizures. 6–24 months: epileptic spasms. Seizure free since then.	Suppression-burst.	Poor eye contact. Global hypotonia, unable to sit (10 years)
**Patient 10**	c.G793A p.A265T	1	Tonic and/or clonic, Multiple seizures daily.	>4 months: myoclonic jerks	Burst of asynchronous spikes and sharp waves. Periods of discontinuity with flatness of the traces without classical suppression burst.	Poor eye contact. Global hypotonia, poor head control, pyramidal signs (6 months)
**Patient 11**	c.A886C p.T296P	1	Tonic, cyanosis, Multiple seizures daily.	0-19 days: multiple seizures. 6-18 m: no seizure. 18 m- 11y: several episodes of CTGC or PS with secondary generalization.	Left or right spikes on a moderately abnormal background.	Walking (18 months). Poor language, autistic features (11 years)
**Patient 12**	c.2318dupG p.C774Lfs*91	4	Partial motor seizure with asymmetric tonic extension of one limb. Bilateral clonic seizures. Apnea.	1 month: seizure free.	Suppression-burst.	Slight peripheral hypertonia (3 months). Good outcome, walking (18 months), normal language (5 years)
**Patient 13**	c.G471A p.W157X	4	Hemi corporeal, left or right.	0-11 months: partial clonic seizures. Then seizure offset.	Poor, discontinuous.	Independent walking (4 y). No language (6.5 y). Normal HC 52.5 cm
**Patient 14**	c.G868A p.G290S	1	Tonic.	Many motor seizures during the neonatal period. 2 m: Seizure stop. AED withdrawn at 4 years.	Asymmetrical suppression-burst with multifocal slow waves, left frontal and right occipital spikes. Periods of generalized flattening.	Sitting (3 y) hand stereotypies. Unable to walk/stand, stereotypies, pyramidal signs. Poor language. Normal HC (16 y).
**Patient 15**	c.C881T p.A294V	8	Myoclonic jerks, Multiple seizures daily.	0-3 months: myoclonic jerks. 3 months: seizure offset. Therapy stopped at 6 months.	Suppression burst.	Sit (2 y). No walking, 2–3 words. Understands simple orders. Strabismus, nystagmus (3 y)
**Patient 16**	c.997C>T. p.R333W	2	Bilateral tonic clonic And right clonic	0-3 y: active epilepsy, motor seizures 3-10 y: seizure free 10-20 y: monthly focal seizures	Slow waves with asynchronous bilateral spikes and intermittent flattening	First steps (18 m). Few words (3 y) Able to read but cannot write, limited communication skills, marked bradypsychia, hand stereotypies (26 y)

*HC *head circumference, *m *months, *y *year.

**Table 2:** Data on initial evaluation and treatment, EEG evolution and brain MRI

	**Term. Clinical examination at birth**	**Treatment during the first month**	**EEG evolution (age)**	**Brain MRI (age)**
**Patient 1**	Full term. Hypotonia. No eye contact. BW: 3000 g HC: 35 cm	PHB VGB	1 m: Continuous with rare posterior spikes and fast rhythms	Day 7: Normal, absence of any signal abnormality. 2 y: Discrete global brain atrophy, thin corpus callosum
			6 m: slow background, rare focal spikes.	
**Patient 2**	34 GW Fetal distress, apnea, movements disorder BW: 2,040 g HC: 30 cm	MDZ, PHB, B6, TPM, VGB	0-7 m: Suppression-burst >7 m: Hypsarythmia	D13: absence of any signal abnormality. 3 m: Absence of signal abnormality
**Patient 3**	Full term Failure. to thrive. Feeding difficulties BW: 3,770 g HC: 37.5 cm	PHB, B6, PHT, VGB, TPM, CLB.	1-6 w: Asynchronous SB. 2-8 m: bilateral Bursts of central spikes 1-6 y: Bursts of rhythmic generalized spikes at 3 Hz	Day 3: T1 bilateral hypersignal of pallida, tegmentum, locus niger, hippocampi. Abnormal ADC in these regions. 2 y: T1 hypersignal of the same structures and diffuse T1 hypersignal of the white matter. Brain atrophy
**Patient 4**	Full term. Normal BW: 3,580 g HC: 37 cm	PHB, PHT, VPA	0-2 m: Suppression-burst. 2–12 m: hypsarythmia. >12 m: Frequent multifocal spikes	Day 7: Normal CT scan 2 y: Thin Corpus Callosum, absence of signal abnormality
**Patient 5**	Full term. Normal BW: 3,240 g HC: 34 cm	PHB, PHT, B6, VGB, VPA	0-2 m: Suppression-burst. 2–6 m: continuous, slow background, multifocal spikes. >6 m: Rare spikes in temporal and occipital lobes	Day 10: no signal abnormality.
**Patient 6**	Full term. Normal BW: 2,790 g HC: 34,5 cm	ND	0-1 m: Suppression-burst. <1 m: Multifocal spikes, slow background	1 m: Normal
**Patient 7**	Full term. Normal BW: 3,180 g, HC: 36 cm	PHB, B6, PHT	0-1 m: Suppression-burst. 1–7 m: Continuous EEG, multifocal spikes 7–24 m: Hypsarythmic pattern. >24 m: Frequent spikes and spike wave in frontal regions	Day 4: Normal, absence of any signal abnormality
**Patient 8**	ND	PHB, PHT	0-2 m: Bursts of multifocal spikes, periods of flatness. >2 m: Multifocal spikes, poor organization	1 y: No structural or signal abnormality.
**Patient 9**	At term. Hypotonia. No eye contact. BW: 3,450 g HC: 35 cm	PB, CZP, VGB	0-2 m: Suppression-burst 2–6 m: Slow background, rare generalized spike waves. 6–12 m: Hypsarythmic pattern. >12 m: Rare asynchronous frontal and temporal spikes	Day 10: Normal, absence of any signal abnormality
**Patient 10**	Full term. No eye contact BW: 3,120 g HC: 33 cm	PB, PHT, TPM, VGB, B6,	0-2 m: discontinuous EEG. >2 m: continuous, slow EEG with rare generalized spikes	Day 5: T1: symmetrical hypersignal of the pallida, caudate nuclei and hippocampi T2: bilateral hypersignal of the parietal occipital white matter
**Patient 11**	Full term. Hypotonia, hyporeactivity, failure to feed	PHB, PHT, VPA.	0-1 m: Left or right spikes on a moderately abnormal background. >1 m: Occipital or temporal spikes with left prominence with progressive migration on the central temporal region	1 m: normal
**Patient 12**	Full term. Normal BW and HC	VGB, CBZ	0-2 m: Suppression-burst. 2–6 m: General slowing of the traces, no spike. 6 m-2 y: Rare spikes in the right central region, Normal background. >2 y: normal traces.	Day 7: T2 hyperintensity of the basal ganglia 2 y: Normal 3y: Normal
**Patient 13**	Full term. Fetal distress. BW, HC: ND	ND	ND	1 m: No structural abormality, no signal change
**Patient 14**	Full term. BW 3,750 g. Poor eye contact, trunk hypotonia with bouts of hypertonia	PHB, VGB, CBZ;	0-4 m: Asymmetrical suppression-burst 4-10 m: Left occipital spikes and slow waves 10 m-3 y: Normal background activity + posterior theta waves, No spike 3 y: Intermittent slow background, no spike >8 y: Normal	3 m: Normal
**Patient 15**	Oligoamnios Born at 30 Weeks (GA) BW: 1580 g HC: 29 cm	PHB, VPA	0-1 m: Suppression-burst >1 m: continuous traces (normal)	2 y: normal
**Patient 16**	Full term Global hypotonia, weak cry	CLN, PHT	0-1 m: absence of physiologic features, slow waves, spikes, brief flattening. 1–6 m: improvement of background activity, some generalized flattening episodes, left occipital slow waves. 6 m – 6 y: slow. background activity, rare spikes. 27 y: bilateral temporal slow waves. 30 y: normal background activity, bilateral fronto-temporal bursts of slow waves, photic stimulation-evoked slow spikes	17 y: slight T2 and FLAIR hyperintensity of thalami.

*GW *gestational week, *HC *Head circumference, *m *month, *Y *year, *PHB *Phenobarbital, *PHT *phenytoine, *VGB *vigabatrin, *TPM* topiramate, *CLN *clonazepam, *VPA *Sodium Valproate, *CBZ *carbamazepine, *CLB* clobazam.

## Conclusions

*KCNQ2* is frequently found mutated de novo in early onset epileptic encephalopathies, especially if the epilepsy begins within the first week of life. Despite relatively stereotyped initial phenotype, the neurological and epileptic outcomes were highly variable, overall severe.

## Competing interests

The authors declare that they have no competing interests.

## Authors’ contributions

MM, SA, CM, NV, AR, BH, MC, AK, CA, GB, DV, MAB, DH, DG, AA, ND, TBV, JP, BI, NG, LV, IA, AJ, PC BC, GL and LV designed the study and interpreted the data. NBK, JSS and CL made the experiments. MM, CM, GL and LV drafted and revised the MS. All authors read and approved the final manuscript.

## References

[B1] Biervert C Schroeder BC Kubisch C Berkovic SF Propping P Jentsch TJ Steinlein OK A potassium channel mutation in neonatal human epilepsy Science 1998 279 403 406 10.1126/science.279.5349.403 9430594

[B2] Charlier C Singh NA Ryan SG Lewis TB Reus BE Leach RJ Leppert M A pore mutation in a novel KQT-like potassium channel gene in an idiopathic epilepsy family [see comments] Nat Genet 1998 18 53 55 10.1038/ng0198-53 9425900

[B3] Singh NA Charlier C Stauffer D DuPont BR Leach RJ Melis R Ronen GM Bjerre I Quattlebaum T Murphy JV McHarg ML Gagnon D Rosales TO Peiffer A Anderson VE Leppert M A novel potassium channel gene, KCNQ2, is mutated in an inherited epilepsy of newborns Nat Genet 1998 18 25 29 10.1038/ng0198-25 9425895

[B4] Plouin P Benign familial neonatal convulsions and benign idiopathic neonatal convulsions Epilepsy: a comprehensive textbook 1997 Philadelphia: Lippincott-Raven 2247 2249

[B5] Bellini G Miceli F Soldovieri MV Miraglia Del Giudice E Coppola G Taglialatela M Pagon RA, Bird TD, Dolan CR KCNQ2 related disorders. 2010 Apr 27 [Updated 2013 Apr 11] GeneReviews™ [Internet] 1993 Seattle (WA): University of Washington, Seattle: Available from: http://www.ncbi.nlm.nih.gov/books/NBK32534/

[B6] Dedek K Fusco L Teloy N Steinlein OK Neonatal convulsions and epileptic encephalopathy in an Italian family with a missense mutation in the fifth transmembrane region of KCNQ2 Epilepsy Res 2003 54 21 27 10.1016/S0920-1211(03)00037-8 12742592

[B7] Weckhuysen S Mandelstam S Suls A Audenaert D Deconinck T Claes LR Deprez L Smets K Hristova D Yordanova I Jordanova A Ceulemans B Jansen A Hasaerts D Roelens F Lagae L Yendle S Stanley T Heron SE Mulley JC Berkovic SF Scheffer IE de Jonghe P KCNQ2 Encephalopathy: emerging phenotype of a neonatal epileptic encephalopathy Ann Neurol 2012 71 15 25 10.1002/ana.22644 22275249

[B8] Saitsu H Kato M Koide A Goto T Fujita T Nishiyama K Tsurusaki Y Doi H Miyake N Hayasaka K Matsumoto N Whole exome sequencing identifies KCNQ2 mutations in ohtahara syndrome Ann Neurol 2012 72 298 300 22926866 10.1002/ana.23620

[B9] Saitsu H Kato M Mizuguchi T Hamada K Osaka H Tohyama J Uruno K Kumada S Nishiyama K Nishimura A Okada I Yoshimura Y Hirai S Kumada T Hayasaka K Fukuda A Ogata K Matsumoto N De novo mutations in the gene encoding STXBP1 (MUNC18-1) cause early infantile epileptic encephalopathy Nat Genet 2008 40 782 788 10.1038/ng.150 18469812

[B10] Kato M Saitoh S Kamei A Shiraishi H Ueda Y Akasaka M Tohyama J Akasaka N Hayasaka K A longer polyalanine expansion mutation in the ARX gene causes early infantile epileptic encephalopathy with suppression-burst pattern (ohtahara syndrome) Am J Hum Genet 2007 81 361 366 10.1086/518903 17668384 PMC1950814

[B11] Miceli F Soldovieri MV Ambrosino P Barrese V Migliore M Cilio MR Taglialatela M Genotype-phenotype correlations in neonatal epilepsies caused by mutations in the voltage sensor of Kv7.2 potassium channel subunits Proc Natl Acad Sci USA 2013 110 4386 91 10.1073/pnas.1216867110 23440208 PMC3600471

